# Hemangioma of the Rib Mimicking Chondrosarcoma: A Report of Two Cases and Literature Review

**DOI:** 10.1155/2021/9996380

**Published:** 2021-09-28

**Authors:** Yusuke Tsuda, Hiroshi Kobayashi, Naohiro Makise, Liuzhe Zhang, Yusuke Shinoda, Tetsuo Ushiku, Sakae Tanaka

**Affiliations:** ^1^Department of Orthopaedic Surgery, The University of Tokyo Hospital, 7-3-1 Hongo, Bunkyo-ku, Tokyo 11386-55, Japan; ^2^Department of Pathology, The University of Tokyo Hospital, 7-3-1 Hongo, Bunkyo-ku, Tokyo 113-8655, Japan

## Abstract

**Cases:**

Case 1 was a 58-year-old man who presented with an incidentally detected, slowly growing mass in the right hypochondrium area. An imaging study showed the mass arising from the 11th rib, with ill-defined margins and cortical destruction. Differential diagnoses included chondrosarcoma and metastatic malignant tumor. Open biopsy was associated with moderate bleeding (300 mL) despite small incision. Microscopic findings showed numerous irregular, dilated, and thin-walled vessels, consistent with the diagnosis of hemangioma of bone, and *en bloc* excision was performed with no surgical complication. Case 2 was a 49-year-old man who presented with an incidentally detected 4th rib mass with calcification on computed tomography scan. Chondrosarcoma was suspected according to imaging features. An open biopsy was considered to have a risk of tumor seeding because the tumor was located behind the scapula. *En bloc* excision of the tumor without biopsy was performed. The pathological findings were consistent with hemangioma of bone.

**Conclusion:**

We reported two cases of rare hemangioma arising from the rib, which mimicked chondrosarcoma. The preoperative diagnosis was challenging, both clinically and radiologically. Because biopsy for hemangioma of the rib is associated with a bleeding risk, the *en bloc* excision without biopsy can be a practical treatment option.

## 1. Introduction

Hemangioma of bone is a benign tumor composed of vascular channels of either small or large caliber [1], and the vertebral bodies are the most commonly affected sites. It accounts for approximately 1% of all bone tumors. Here, we present two cases of rare hemangioma arising from the rib, which mimicked chondrosarcoma. The previously reported clinical features and imaging findings were reviewed, and we also discussed the treatment and diagnostic strategies for rib hemangioma, including biopsy.

## 2. Case Presentation

### 2.1. Case 1

**Figure 1:**
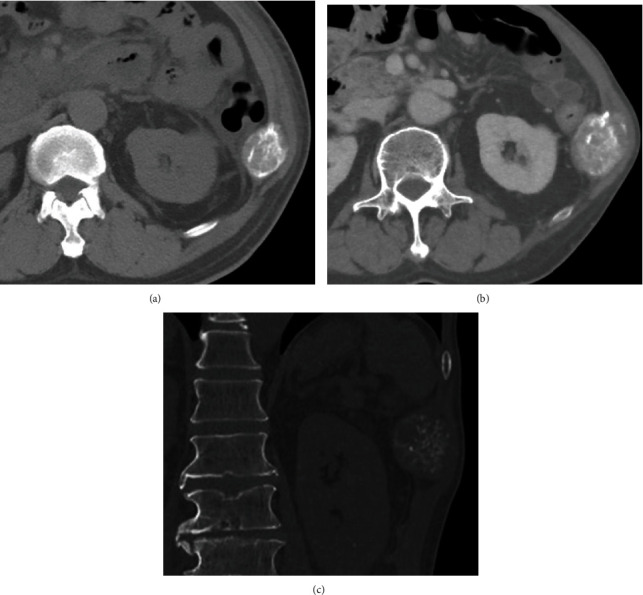
(a) Computed tomography (CT) image of the tumor showing cortical destruction and calcification in the 11th rib. (b) The tumor slowly grew at three-year intervals. (c) Calcification resembled the ring and arc calcifications observed in chondrosarcoma on CT.

**Figure 2:**
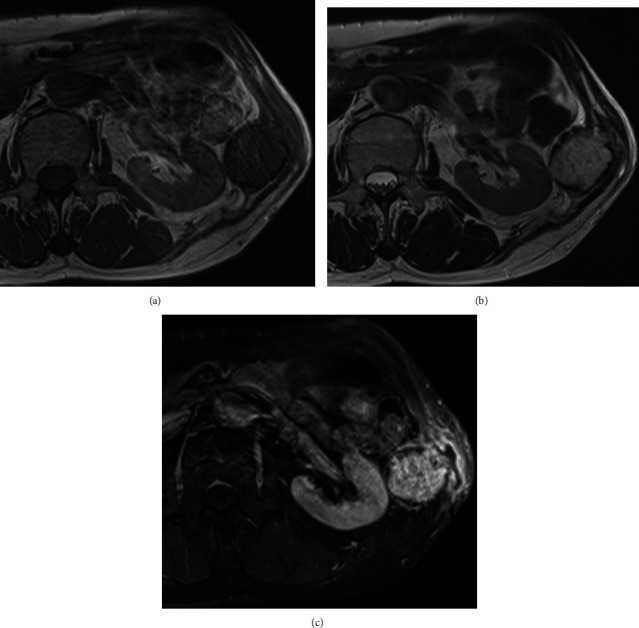
Magnetic resonance imaging showing the tumor in the 11th rib. (a) The mass was isointense relative to skeletal muscle on T1-weighted images. (b) The mass was hyperintense relative to skeletal muscle on T2-weighted images. (c) The mass showed enhancement on gadolinium-enhanced, fat-saturation T1-weighted images.

**Figure 3:**
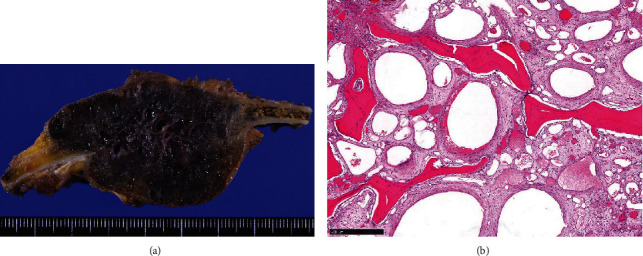
(a) Gross appearance of the resected specimen. Numerous vessels were identified. (b) The histopathology of the lesion showed that the tumor was comprised of numerous irregular, dilated, and thin-walled vessels lined with a single layer of flattened endothelial cells, without cytologic atypia, consistent with the findings of hemangioma. The coarse trabecular bones were observed.

A 58-year-old man was referred for the evaluation of a slowly expanding bone mass arising from the right 11th rib. The lesion was incidentally detected during treatment for pyogenic spondylitis and infective endocarditis on computed tomography (CT) (Figures 1(a)–1(c)). He was asymptomatic and did not report pain or weight loss. He had received anticoagulant (warfarin potassium) and antiplatelet therapy (aspirin) after mitral valve replacement for infective endocarditis associated with atrial fibrillation. X-ray and CT examination showed a lytic lesion with cortical bone destruction in the 11th rib. Calcification or coarse trabeculae was detected in the mass. The tumor was 5 cm in size. Magnetic resonance imaging (MRI) demonstrated a well-circumscribed, lobulated mass, which appeared isointense relative to skeletal muscle on T1-weighted images, presented high-signal intensity on T2-weighted images and enhancement on gadolinium-enhanced T1-weighted images (Figures 2(a)–2(c)). Chondrosarcoma and metastatic tumors were suspected based on the clinical presentation and imaging findings although the tumor showed slow growth. A CT-guided biopsy was unsuccessful due to bleeding during the procedure. Warfarin treatment was bridged to heparin one week before open biopsy and discontinued 12 hours before the open biopsy. APTT was within normal range (24.4 seconds) immediately before the open biopsy. Although long manual compression and hemostatic agents were applied during the open biopsy with small surgical incision, the patient experienced moderate bleeding (300 mL). Microscopically, the tumor comprised numerous irregular, dilated, and thin-walled vessels lined with a single layer of flattened endothelial cells without cytologic atypia, indicating hemangioma. CD31 immunostaining was positive for tumor cells. The patient underwent *en bloc* tumor excision, including the adjacent 11th rib, with no surgical complications. Pathological findings were consistent with the features of hemangioma of bone (Figures 3(a) and 3(b)). At the 3-month postoperative follow-up visit, no local tumor recurrence was observed.

### 2.2. Case 2

**Figure 4:**
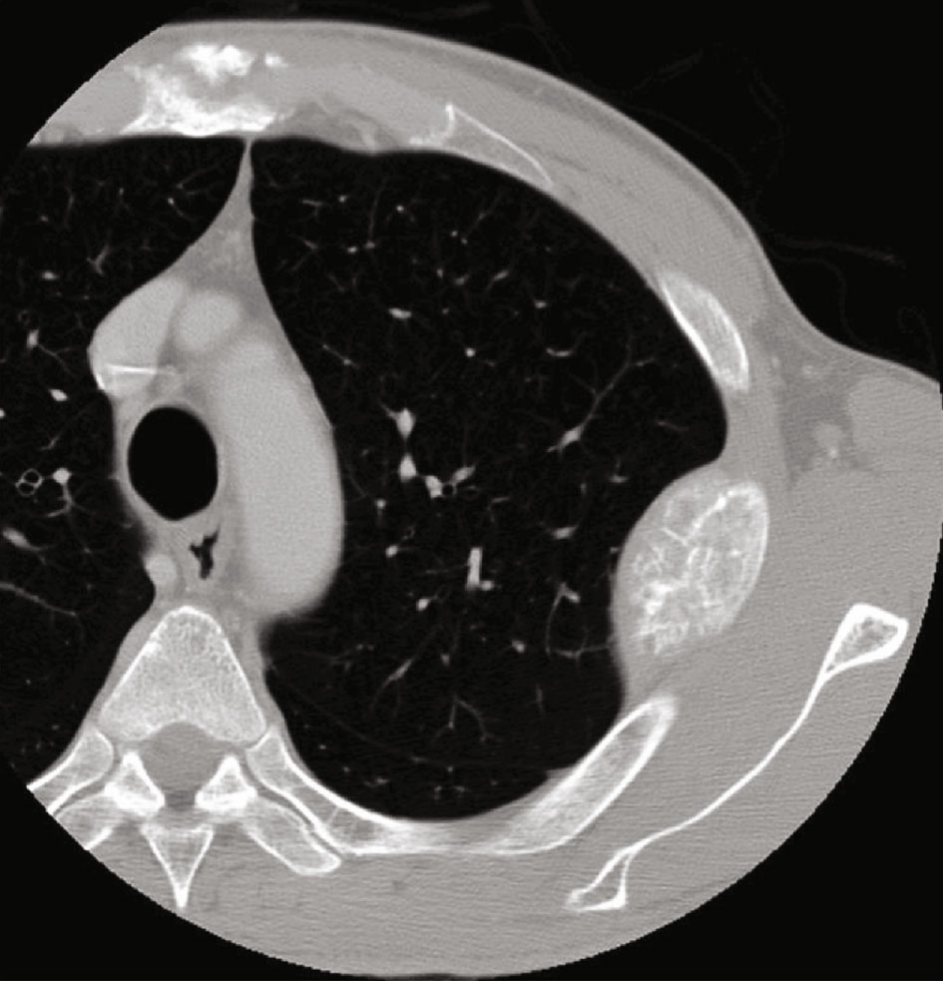
Computed tomography showing a tumor with cortical destruction and honeycomb-like calcifications in the 4th rib.

**Figure 5:**
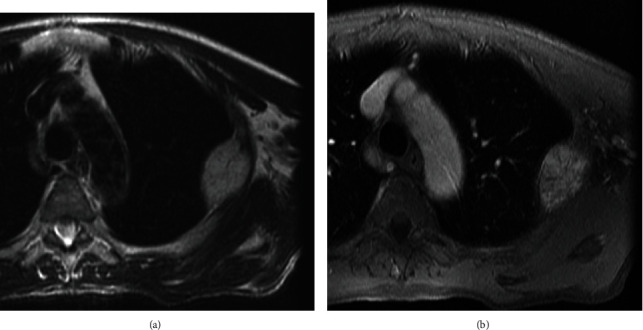
Magnetic resonance imaging showing the tumor in the 4th rib. (a) The mass was hyperintense on T2-weighted images. (b) The mass showed enhancement on gadolinium-enhanced, fat-saturation T1-weighted images.

A 49-year-old man presented with a 4th rib mass that was incidentally detected on chest X-ray during a regular checkup. X-ray and CT imaging showed a mass with honeycomb-like calcifications in the 4th rib (Figure 4). MRI demonstrated a corresponding mass, which appeared isointense relative to skeletal muscle on T1-weighted images, with high-signal intensity on T2-weighted images, and enhancement on gadolinium-enhanced T1-weighted images (Figures 5(a) and 5(b)). Chondrosarcoma was suspected based on the imaging features. No open biopsy was performed due to tumor seeding risk as the tumor was located behind the scapula. The *en bloc* excision of the tumor, along with the adjacent 4th rib, was performed. The pathological findings were consistent with hemangioma of bone. No surgical complications were observed. At the 18-year postoperative follow-up visit, no local tumor recurrence was detected. Both patients were informed that the data from their cases would be submitted for publication, and they provided their informed consent.

## 3. Discussion

The majority of hemangiomas of bone involve vertebral bodies, followed by the craniofacial skeleton and long bones. Hemangioma of the rib is rare, with only 33 cases of rib hemangiomas, including our two cases, described in the English literature to date [2–9]. In the literature and our cases, the median patient age was 50 years (range, 11–76 years). The tumors ranged from 3 to 16 cm in size (median, 6 cm), were predominantly asymptomatic (20 of 33, 61%), and were typically detected incidentally. Many rib hemangiomas (25 of 33, 76%) showed osteolytic changes on X-ray or CT, which resembled other malignancies, including chondrosarcoma [5–7].

On CT, hemangioma of the rib presents as an expansile, lytic bone mass with a honeycomb appearance in the medullary cavity and a thin bony cortex either with or without cortical destruction [2]. On MRI, bone hemangioma appears hypointense on T1-weighted images and hyperintense on T2-weighted images. In our case, the mass demonstrated cortical destruction, and the bony structures in Case 1 were similar to the rings and arcs of calcification which was associated with chondrosarcoma. The observed hyperintensity on T2-weighted MRI also resembled chondrosarcoma in both cases. Common malignant tumors of the rib include metastatic tumors, myeloma, and chondrosarcoma [8]. Therefore, we considered chondrosarcoma as the differential diagnosis. Diagnosis without pathological findings was challenging, both clinically and radiologically. A previous report has suggested that delayed-phase contrast-enhanced MRI can be helpful for distinguishing hemangioma of the rib from chondrosarcoma [10]. Diffuse mass enhancement on delayed-phase contrast-enhanced MRI was an atypical finding because conventional chondrosarcoma with abundant chondroid matrix displays low degrees of enhancement on delayed-phase contrast-enhanced MRI. In the case of rib masses with septum- or honeycomb-like calcifications on CT images and marked hyperintensity on T2-weighted images, the internal enhancement observed on delayed-phase contrast-enhanced MRI is indicative of a rich fibrous stroma or vessels, which represents an important clue to suggest the diagnosis of hemangioma of the rib [10]. Although Fluorine-18 2-fluoro-2-deoxy-D-glucose (FDG) positron emission tomography (PET) is useful to obtain more information about benign and malignant tumors, hemangioma of the rib showed a relatively high maximum standard uptake value (SUVmax) of 2.2–6.7 [4]. Therefore, FDG-PET may not provide useful information for distinguishing rib hemangioma from malignant tumors including chondrosarcoma.

For Case 1, the preoperative open biopsy was associated with moderate bleeding despite small incision, whereas the biopsy was avoided to reduce the risk of tumor seeding in Case 2. Some authors have stated that biopsy should be avoided due to the tumor-seeding risk through the biopsy tract and bleeding risk [11, 12], whereas others have emphasized the importance of a biopsy to obtain a definitive diagnosis [13]. Critical bleeding and hematoma formation were reported in some patients who underwent biopsies for rib hemangioma [2, 14]. We believe that *en bloc* tumor excision without biopsy represents a practical treatment option in cases with tumor seeding or bleeding risk to avoid the complications due to biopsy. Moreover, when the degree of dysfunction after *en bloc* excision is minimal and the probability of high-grade sarcoma or bone metastasis is low through clinical examination and systemic workup, the biopsy could be omitted. Curettage for vascular tumors also has the risk of large bleeding events during the surgical procedure. Therefore, *en bloc* excision is typically performed for rib hemangioma [2–9]. On the basis of previous report [15], *en bloc* excision with at least a 2 mm margin is advisable for obtaining optimal oncological outcomes in patients without biopsy because this margin was considered a safe margin for high-grade chondrosarcoma.

In conclusion, we reported two rare cases of rib hemangioma mimicking chondrosarcoma. *En bloc* excision without biopsy can be a practical treatment option to avoid bleeding or tumor seeding risks. Delayed-phase contrast-enhanced MRI may be helpful for differential diagnosis.
